# The benefits of exercise training in interstitial lung disease: protocol for a multicentre randomised controlled trial

**DOI:** 10.1186/1471-2466-13-8

**Published:** 2013-02-01

**Authors:** Leona Dowman, Christine F McDonald, Catherine Hill, Annemarie Lee, Kathryn Barker, Claire Boote, Ian Glaspole, Nicole Goh, Annemarie Southcott, Angela Burge, Rebecca Ndongo, Alicia Martin, Anne E Holland

**Affiliations:** 1 Department of Physiotherapy, Austin Health, Melbourne, Australia; 2 Department of Physiotherapy, La Trobe University, Melbourne, Australia; 3 Institute for Breathing and Sleep, Melbourne, Australia; 4 Department of Respiratory & Sleep Medicine, Austin Health, Melbourne, Australia; 5 The University of Melbourne, Melbourne, Australia; 6 Department of Physiotherapy, Alfred Health, Melbourne, Australia; 7 Department of Physiotherapy, Western Health, Melbourne, Australia; 8 Allergy, Immunology & Respiratory Medicine Department, Alfred Health, Melbourne, Australia; 9 Department of Respiratory & Sleep Disorders Medicine, Western Health, Melbourne, Australia

**Keywords:** Interstitial lung diseases, Diffuse parenchymal lung diseases, Idiopathic pulmonary fibrosis, Idiopathic interstitial pneumonias, Asbestosis, Sarcoidosis, Hypersensitivity pneumonitis, Connective tissue diseases, Exercise, Rehabilitation

## Abstract

**Background:**

Interstitial lung disease encompasses a diverse group of chronic lung conditions characterised by distressing dyspnoea, fatigue, reduced exercise tolerance and poor health-related quality of life. Exercise training is one of the few treatments to induce positive changes in exercise tolerance and symptoms, however there is marked variability in response. The aetiology and severity of interstitial lung disease may influence the response to treatment. The aims of this project are to establish the impact of exercise training across the range of disease severity and to identify whether there is an optimal time for patients with interstitial lung disease to receive exercise training.

**Methods/Design:**

One hundred and sixteen participants with interstitial lung disease recruited from three tertiary institutions will be randomised to either an exercise training group (supervised exercise training twice weekly for eight weeks) or a usual care group (weekly telephone support). The 6-minute walk distance, peripheral muscle strength, health-related quality of life, dyspnoea, anxiety and depression will be measured by a blinded assessor at baseline, immediately following the intervention and at six months following the intervention. The primary outcome will be change in 6-minute walk distance following the intervention, with planned subgroup analyses for participants with idiopathic pulmonary fibrosis, dust-related interstitial lung disease and connective-tissue related interstitial lung disease. The effects of disease severity on outcomes will be evaluated using important markers of disease severity and survival, such as forced vital capacity, carbon monoxide transfer factor and pulmonary hypertension.

**Discussion:**

This trial will provide certainty regarding the role of exercise training in interstitial lung disease and will identify at what time point within the disease process this treatment is most effective. The results from this study will inform and optimise the clinical management of people with interstitial lung disease.

**Trial registration:**

Australian New Zealand Clinical Trials Registry ACTRN12611000416998

## Background

The interstitial lung diseases (ILDs) are a disabling and diverse group of chronic lung conditions that have been broadly classified into four groups: ILD of known cause such as occupational or environmental exposures and/or collagen vascular disease; granulomatous ILD such as sarcoidosis; idiopathic interstitial pneumonias including idiopathic pulmonary fibrosis (IPF) and nonspecific interstitial pneumonia (NSIP); and other rare forms of ILD including lymphangioleiomyomatosis, pulmonary Langerhans’ cell histiocytosis/histiocytosis X, and eosinophilic pneumonia [1]. Many ILDs are characterised by the development of irreversible and progressive interstitial fibrosis of the lung parenchyma [2] resulting in altered respiratory mechanics, impaired gas exchange, reduced exercise capacity and dyspnoea on exertion [3-5]. Skeletal muscle dysfunction and weakness may occur, leading to worsening exercise capacity and increasing symptoms [2,6,7]. Health-related quality of life (HRQoL) is frequently markedly reduced and those with the greatest exercise limitation report the worst quality of life [8]. As disease progresses, severe hypoxemia and pulmonary hypertension may develop [9,10], with patients often becoming dependent on supplemental oxygen.

The classification of the ILDs has been the subject of criticism, due to its failure to reflect the marked heterogeneity in clinical course within disease subgroups. Idiopathic pulmonary fibrosis (IPF), the best characterized of the ILDs, is largely a fatally progressive disease with a median survival of 3–5 years [11]. The prognosis of NSIP is more variable, and, although a minority of patients may have an accelerated decline similar to IPF [10], survival is generally significantly longer than in IPF. Dust and connective tissue disease- related ILD may be associated with a better overall survival rate but can result in significant and progressive morbidity over many years [12,13]. Prognosis of sarcoidosis is again variable and difficult to predict with stabilisation or improvement in some patients and the development of progressive pulmonary fibrosis in others [10,14].

Few treatments have demonstrated improvements in either HRQoL or community functioning for any of the ILDs [15,16]. In IPF, the most common and most lethal ILD, the options for pharmacological treatment are very limited [11]. Therapies that can improve dyspnoea, fatigue, exercise capacity and quality of life are highly sought after in ILD [16]. Exercise is one of the few treatments to show positive changes in functional capacity and symptoms. We have previously shown that exercise training could significantly improve exercise capacity and reduce dyspnoea and fatigue symptoms in patients with ILD of varying aetiology [17]. Nishiyama et al. found similar positive effects from exercise training in patients with IPF only [18]. Additionally, several observational studies evaluating the benefits of pulmonary rehabilitation, of which exercise training is an essential component, demonstrated statistically and clinically significant improvements in functional capacity, dyspnoea and HRQoL in patients with ILD of varying aetiology [19-23]. Despite these promising outcomes exercise training is not yet widely recommended for people with ILD. Only weak recommendations regarding exercise training are provided in the most recent clinical guidelines for the diagnosis and management of IPF [11] and ILD [10].

Uncertainty remains regarding the clinical relevance of exercise training across the entire range of ILDs. Patients with IPF appear to have smaller gains in functional capacity than those with ILD of other aetiology [24]. This raises the possibility that some forms of ILD may respond to exercise training better than others. Common manifestations of ILD, such as exercise induced hypoxia and pulmonary hypertension [2,9], may also affect the improvements that may be achieved. Hypoxaemia impairs maximal exercise performance [3] and pulmonary hypertension in ILD is associated with considerably reduced exercise capacity and greater exercise limitation [25-27]. In an uncontrolled study evaluating the relationship between response to exercise and disease aetiology and severity in forty-four subjects with ILD of varying aetiology, less severe lung function, less oxyhaemoglobin desaturation and less pulmonary hypertension were associated with greater improvement in functional capacity in patients with IPF [28]. This relationship persisted at six months, suggesting that those with less advanced IPF may be able to achieve sustained benefits from exercise training. This relationship was not seen in subjects with other ILDs. It is therefore possible that the timing of exercise training may be important for patients with IPF, whereas patients with other forms of ILD may benefit regardless of disease severity.

In order for exercise training to be widely adopted in clinical practice, clinicians require more information regarding its role across the disease spectrum. The aims of this study are 1) to establish the impact of exercise training on ILDs of different aetiology and severity and 2) to identify whether there is an optimal timing for exercise training to achieve maximal benefit. We hypothesise that exercise training will be effective regardless of disease severity in patients with non-IPF related ILD, whereas in patients with IPF, the response to exercise training will be greatest in those with less severe disease.

## Methods

### Study design

This multi-centre randomised controlled trial will be conducted at Alfred Health, Austin Health and Western Health, Melbourne, Australia.

### Participants

Participants with a documented diagnosis of ILD will be recruited for this study from the Departments of Respiratory and Sleep Medicine at Alfred, Austin and Western Health. The diagnosis of ILD will be made according to established criteria. In IPF, the diagnostic criteria will be consistent with those outlined in the International Consensus Statement [11]. A surgical lung biopsy will not be required for entry into the study as it has been demonstrated that clinical and radiologic data are sufficient to distinguish between IPF and other ILDs in the hands of experienced clinicians [29]. Diagnosis of connective tissue disease will be made according to the rheumatological criteria for that disease; ILD in this setting will be diagnosed according to clinical/radiologic and lung function criteria, with lung biopsy in atypical cases. Dust-related ILD will be confirmed according to accepted criteria that include significant exposure to an agent recognised to cause ILD and radiological confirmation on high resolution computed tomography of the chest, as determined by independent radiologists.

Participants must be clinically stable, ambulant, and suffer from dyspnoea on exertion despite maximal appropriate medical treatment. Participants will be excluded if they 1) have a concurrent and predominant diagnosis of another significant respiratory disorder (for example: asthma, chronic obstructive pulmonary disease [COPD], bronchiectasis, cystic fibrosis, or lung carcinoma) which is the primary cause of their symptoms; 2) have a history of syncope on exertion; 3) are too unwell to attend the hospital for exercise training; 4) have any other co-morbidities, such as severe orthopaedic or neurological deficits or unstable cardiac disease which would prevent exercise training; 5) have participated in a pulmonary rehabilitation program within the previous 12 months.

### Sample size

One hundred and sixteen participants will be required to detect a significant difference in the primary outcome measure of change in functional exercise capacity (6-minute walk distance [6MWD]). This is based on the 80% probability of detecting a difference in the change in 6MWD between the intervention and control group using data from our previous randomised control trial [17] and Cochrane review [24]. Our sample size calculation of 116 has been powered to include the required number of participants in the three most commonly observed ILD subgroups: IPF, dust-related ILD and connective tissue disease-related ILD. To detect a true difference in the change in 6MWD in subjects with IPF, a total of 72 subjects, 36 in each group, is required. This is based on the lower limit of the range for the minimal important difference (MID) of 29m [30] with a standard deviation (SD) of 43m. To detect a true difference in the change in 6MWD between groups using the upper limit of the MID of 34m [30] with SD 43m, a total of 54 subjects, 27 in each group, would be required. In subjects with dust-related ILD, a total of 22 subjects, 11 in each group, is required. This assumes that the true difference between groups is 52m with SD of 40m. In subjects with connective tissue disease-related ILD, 22 subjects, 11 in each group, is required, assuming a difference of 38m with SD 30m.

Data from our previous study [28] indicate that to detect a relationship between carbon monoxide transfer factor (TLCO) and change in 6MWD following pulmonary rehabilitation with 80% power will require 31 subjects in the pulmonary rehabilitation group. This assumes that the true change in 6MWD is 15 meters for each 10% change in baseline percent predicted TLCO. To detect a relationship between degree of pulmonary hypertension and change in 6MWD with 80% power will require 35 subjects in the pulmonary rehabilitation group. This assumes that the true change in 6MWD is 17 meters for each 10mmHg change in baseline right ventricular systolic pressure.

### Recruitment and randomisation

**Figure 1:**
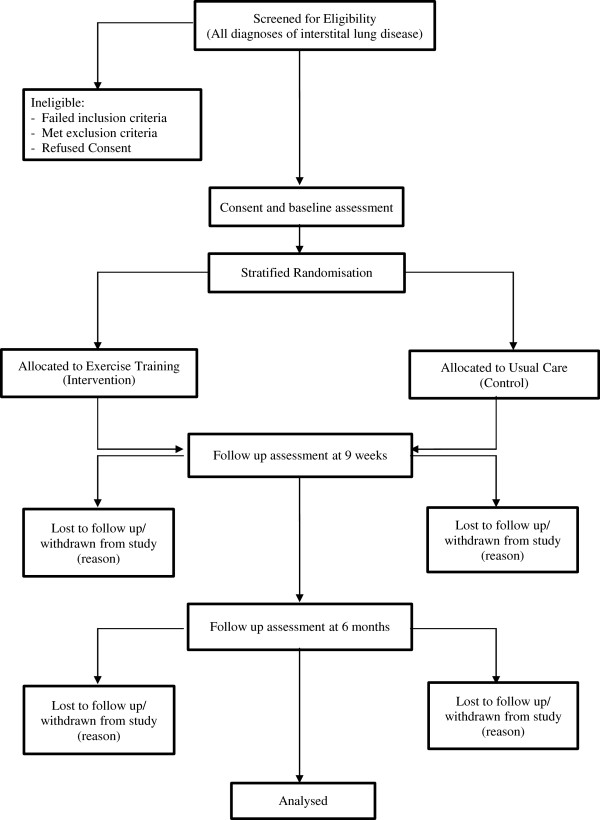
Flow of patients through the study.

The flow of participants through the study will reflect the recommendations from the Consolidated Standards of Reporting Trials statement [31] and is outlined in Figure 1. Participants will be identified at their regular outpatient clinic appointments to the Departments of Respiratory and Sleep Medicine at Alfred Health, Austin Health and Western Health. Eligible participants will be approached by the researchers who will explain the study. Participants will receive written and verbal information about the study and written consent will be obtained from all participants. The Human Research Ethics Committees of Alfred Health, Austin Health, Western Health and La Trobe University approved the study. The study protocol has been registered with the Australian New Zealand Clinical Trials Registry (ACTRN12611000416998).

Randomisation will be stratified according to the three subgroups IPF, dust-related ILD and connective tissue disease-related ILD. This will ensure that all subgroups of ILD are evenly distributed between the intervention and control groups. The randomisation will also be stratified for disease severity according to TLCO <or ≥40% to ensure that those with severe disease are evenly distributed between the intervention and control groups. A set of permuted blocks will be generated for each of the following subgroups: dust-related ILD, connective tissue disease-related ILD, IPF with TLCO < 40% and IPF with TLCO ≥ 40%. The random allocation sequence will be generated using a computer generated random number list. Concealment of group allocation will be achieved by giving the responsibility for allocation sequence generation and group allocation to a researcher independent of the study and its investigators. The group allocation will be kept in sealed, opaque envelopes in a central location. Following the baseline assessment, participants will be randomly allocated to either the exercise training group or to a control group by a researcher, who is not involved in the recruitment or assessment of the participants or the execution of the intervention, by opening the sealed opaque envelope.

### Intervention

#### Exercise training group

The exercise training group will undergo a twice-weekly supervised exercise training program at the institution where they were recruited for a period of 8 weeks. The exercise program will follow the pulmonary rehabilitation guidelines for exercise prescription in chronic lung disease [32]. Each session will consist of 30 minutes of aerobic exercise plus upper and lower limb resistance training exercises. The aerobic exercise component will comprise of 15 minutes each of stationary cycling and walking, either on the treadmill or along a corridor. The initial walking intensity will be set at a speed that is 80% of the peak walking speed (km/hr) achieved on the 6-minute walk test (6MWT). The initial intensity of the stationary cycling will be prescribed at 70% of their maximum work rate estimated from their 6MWT [33] and will be adjusted to elicit a rating of perceived exertion (RPE) of 12–14 on the 6–20 Borg scale and a dyspnoea score of 3–4 on the modified Borg scale [34]. The duration of exercise on each modality will be adjusted if the participant has a co-morbidity that limits their capability on one specific modality, however the total exercise time will remain at 30 minutes. Interval training will be used for those participants who are unable to tolerate continuous exercise. The resistance program will comprise three lower limb and four upper limb dumbbell exercises. The initial load will correspond to 10-12RM (repetition maximum), that is, a weight that can be lifted correctly and comfortably at least 10 times, but not more than 12 times and elicits a RPE of 12–14 on the 6–20 Borg scale [35,36]. All exercise modalities will be progressed regularly by an experienced exercise physiologist or physiotherapist to maintain dyspnoea and fatigue scores of 3–4 and a RPE score of 12–14. Supplemental oxygen will be provided during training if SpO_2_ on room air is <88% whilst exercising and will be titrated to maintain a SpO_2_ ≥90%.

Once the participant is safely established on a supervised exercise regimen, an unsupervised home exercise program will be prescribed as per current Pulmonary Rehabilitation guidelines [37] to achieve three additional home-based exercise sessions per week. Participants will be educated on how to monitor their symptoms and their level of exertion at home and will be instructed to exercise at an intensity similar to that achieved in the supervised sessions. Participants will record their exercise session in an exercise diary and this diary will be reviewed weekly by the supervising clinician. At the conclusion of the 8-week program, participants will be instructed to continue with their home exercise program four to five times per week thereafter [32,38,39]. Attendance at 12 out of 16 sessions will be considered completion of the intervention.

#### Usual care group

Participants randomised to the usual care group will not undergo any supervised exercise training and will not receive any recommendations regarding exercise training or physical activity. These participants will be contacted once weekly by telephone for the duration of the 8 week intervention period to provide general support and health advice and to answer any queries or concerns the participants may have. These phone calls will be conducted according to a standardised script. This is a commonly used control for exercise training interventions and was used in our earlier randomised controlled trial in ILD [17]. The participants in the usual care group will be offered exercise training at the conclusion of the 6 month follow-up period.

#### Outcome measures

Outcome measures will be collected at baseline, upon completion of the intervention period (nine weeks) and at six months following completion of the intervention (Figure 1). A six month follow-up period is the longest we consider to be clinically feasible without excessive loss of participants due to clinical decline and death. At baseline, data collection will include age, gender, body mass index, past medical history, smoking history, use of oxygen therapy, current pharmological treatment and all of the following outcome measures.

#### Primary outcome measure

1. Change in functional exercise capacity will be measured with 6MWT according to standardised criteria [40]. Two tests will be conducted separated by a 30-minute rest period and the best result recorded. Supplemental oxygen will be used during both 6MWTs in participants who already have exertional oxygen or for those who have resting SpO_2_ <88%. Supplemental oxygen will be used at a flow rate of 4L.min^-1^ for the second test if SpO_2_ <85% during the first 6MWT [41-43]. Follow up tests will be conducted on the same oxygen flow rate. The primary outcome is change in 6MWD from baseline to nine weeks. Six-minute walk distance has been shown to correlate strongly with maximum exercise capacity (VO_2_peak) in IPF and has shown responsiveness to change following pulmonary rehabilitation in IPF [18] and ILD [17].

#### Secondary outcome measures

1. Peripheral muscle strength will be assessed using a hand held dynamometer (Commander Power track II, JTech Medical, Utah, USA). Three maximal isometric contractions of the elbow flexors and knee extensors on the dominant side will be tested. Skeletal muscle weakness, in particular quadriceps weakness, has been shown to correlate strongly with reduced exercise tolerance and exercise capacity in patients with ILD [18,44] and was found to be an independent predictor of exercise capacity at peak exercise in patients with IPF [18]. This measure will assess the contribution of muscle strength changes to changes in exercise tolerance.

2. Health-related Quality of Life (HRQoL) will be measured using the Chronic Respiratory Disease Questionnaire (CRQ), and St George Respiratory Questionnaire idiopathic pulmonary fibrosis specific version (SGRQ-I). The CRQ has been validated previously in ILD [8] and has demonstrated improvements following exercise training in ILD [17]. The SGRQ-I is designed to be more responsive in patients with IPF than the original St George respiratory questionnaire (SGRQ) and has similar psychometric properties to the original SGRQ [45].

3. Dyspnoea will be measured using the University of California San Diego Shortness of Breath Questionnaire (UCSD SOBQ) and the Modified Medical Research Council dyspnoea scale (MMRC). The UCSD SOBQ comprises 24 items that assess dyspnoea over the preceding week and is a reliable and valid instrument used to assess dyspnoea associated with Activities of Daily Living (ADL)s in patients with chronic lung disease [46-48]. The MMRC is a valid measure of breathlessness and symptom severity in ILD [49,50].

4. Anxiety and Depression will be measured using the Hospital Anxiety and Depression Scale (HADS). The HADS has been designed to detect and measure the severity of anxiety and depression and has been shown to be a reliable instrument in evaluating anxiety and depression in IPF and ILD [51,52].

5. The Global Rating of Change Scale will be used to assess the participants’ self-perceived improvement or deterioration over time. The Global rating of change scale involves asking the participant whether there has been any change in their symptoms or walking ability since their commencement in the study [53]. Participants can answer either ‘worse’, ‘about the same’ or ‘better’. If subjects state that they are worse or better they are asked to grade how much worse or better on a Likert scale from one to seven. The global rating of change has been used to establish the minimal important difference for the 6MWD in people with ILD [30].

At 9 weeks and at 6 months follow up participants will undergo repeat measurements of the 6MWT, peripheral muscle strength, CRQ, SGRQ-I, UCSD SOBQ, MMRC, HADS and Global Rating of Change to evaluate the immediate and long term effects of exercise training. An independent assessor, blinded to group allocation, will perform all outcome assessments.

#### Classification of disease severity

1. Respiratory function testing will be performed in accordance with the American Thoracic Society guidelines [54] to quantify disease severity and to assess any clinical change in respiratory function over time. Standard spirometric measures will include forced vital capacity (FVC) and forced expiratory volume in one second (FEV_1_) and will be performed at baseline and at six months follow up. Carbon monoxide transfer factor will be measured at baseline and at six months follow up. Static lung volumes measured via plethysmography will be measured at baseline only and will include total lung capacity (TLC), functional residual capacity (FRC) and residual volume (RV).

2. Pulmonary Hypertension will be assessed by a trans-thoracic echocardiogram. Pulmonary hypertension is a common complication of ILD [4,9] and patients with concomitant pulmonary hypertension are likely to have greater exercise impairment.

The respiratory function tests and transthoracic echocardiogram will be performed by routine clinical personnel at the treating hospital and they will be unaware of the group allocation.

#### Statistical analysis

Data will be analysed using intention-to-treat principles, with inclusion of all available data regardless of whether the intervention is completed. The response of exercise training and control groups will be compared for change in exercise and HRQoL variables using linear mixed model analyses. Planned subgroup analyses will be conducted for participants with IPF, dust-related ILD and connective tissue-related ILD. Multiple regression analysis will be undertaken to establish which subjects respond best to exercise training and when this treatment should be offered, with change in 6MWD following exercise training as the dependent variable. Baseline demographic and physiological variables such as age; gender; disease aetiology; percent predicted TLC; percent predicted FVC; TLCO; pulmonary artery pressure; and the extent of oxyhaemoglobin desaturation during exercise will be used as predictors.

## Discussion

Interstitial lung disease represents a heterogeneous group of chronic, disabling lung disorders [55] which are associated with significant dyspnoea and fatigue, reduced exercise capacity and diminished quality of life [2]. The ILDs are an important cause of respiratory morbidity and mortality across the globe however treatment options for people with ILD are extremely limited. Exercise training is a simple intervention that has the potential to impact outcomes that are of utmost importance to patients [2,16]. Currently exercise training has not been made widely available to patients with ILD, due to variability in outcomes and doubts regarding its efficacy across the spectrum of disease. There is little evidence regarding which individuals with ILD should receive exercise and what is the best timing for exercise training to occur. The type and severity of ILD may be important determinants. It is possible that people with IPF may receive greater benefits if exercise training is undertaken earlier in their disease course and people with other ILDs will receive benefits regardless of the severity of their disease. However there is no robust, adequately powered evidence to confirm this position.

The most recent published guidelines on IPF [11] provide only a weak recommendation for pulmonary rehabilitation as part of managing IPF due to low quality of evidence concerning the benefit of pulmonary rehabilitation in this particular form of ILD. Although they indicate there is moderate quality data demonstrating improvement in functional status and patient-centered outcome, uncertainty still remains regarding duration of benefit and further research is needed to impact on the strength of this recommendation. Similarly the Interstitial Lung Disease Guidelines [10] ascribe a low level of evidence to pulmonary rehabilitation, indicating that there is need for more information before pulmonary rehabilitation can be confidently adopted as a recommended treatment for all ILD patients.

This study has been designed to define the role and impact of exercise training in ILD across the range of disease severity and aetiology and to identify whether an optimal time exists during which exercise training should take place in order to ensure that maximal benefit can be obtained. This study will provide patients and clinicians with certainty regarding the role of exercise training as well as the magnitude and duration of expected benefits. If this trial provides evidence of benefit, it will provide a scientific rationale for pulmonary rehabilitation to be considered standard care for people with ILD.

## Abbreviations

ADLs: Activities of Daily Living; COPD: Chronic obstructive pulmonary disease; CRQ: Chronic Respiratory Questionnaire; FEV1: Forced expiratory volume in one second; FRC: Functional residual capacity; FVC: Forced vital capacity; HADS: Hospital Anxiety and Depression Scale; HRQoL: Health related quality of life; ILD: Interstitial Lung Disease; IPF: Idiopathic Pulmonary Fibrosis; MID: Minimal important Difference; MMRC: Modified Medical Research Council; RM: Repetition Maximum; RPE: Rating of perceived exertion; RV: Residual Volume; SGRQ-I: St George Respiratory Questionnaire idiopathic pulmonary fibrosis specific version; TLC: Total lung capacity; TLCO: Carbon monoxide transfer factor; UCSD SOBQ: University of California San Diego Shortness of Breath Questionnaire; 6MWD: Six-minute walk distance; 6MWT: Six-minute walk test.

## Competing interests

The authors declare that they have no competing interests.

## Authors’ contributions

LD, AH, CM, CH, IG and NG designed the trial protocol. LD, AH, CM, CH, KB, CB, IG, NG, AS and AM procured the study funding. LD drafted the manuscript and AH, CM, CH, AL, KB, CB, IG, NG, AS, AB, AM, and RN contributed to the manuscript. All authors read and approved the final manuscript.

## Pre-publication history

The pre-publication history for this paper can be accessed here:

http://www.biomedcentral.com/1471-2466/13/8/prepub
